# Characterization of Oxygen Levels in an Uninfected and Infected Human Blood-Cerebrospinal-Fluid-Barrier Model

**DOI:** 10.3390/cells11010151

**Published:** 2022-01-04

**Authors:** Alexander Martens, Nicole de Buhr, Hiroshi Ishikawa, Horst Schroten, Maren von Köckritz-Blickwede

**Affiliations:** 1 Department of Biochemistry, University of Veterinary Medicine Hannover, 30559 Hannover, Germany; alexander.martens@tiho-hannover.de; 2 Research Center for Emerging Infections and Zoonoses (RIZ), University of Veterinary Medicine Hannover, 30559 Hannover, Germany; 3 Laboratory of Clinical Regenerative Medicine, Department of Neurosurgery, University of Tsukuba, Tsukuba-City, Inaraki 305-8575, Japan; ishi-hiro.crm@md.tsukuba.ac.jp; 4 Department of Pediatrics, Pediatric Infectious Diseases, Medical Faculty Mannheim, Heidelberg University, 68167 Mannheim, Germany; Horst.Schroten@umm.de

**Keywords:** blood-cerebrospinal-fluid-barrier (BCSFB), *Streptococcus suis*, neutrophils, oxygen, hypoxia, physioxia, meningitis

## Abstract

The host–pathogen interaction during meningitis can be investigated with blood-cerebrospinal-fluid-barrier (BCSFB) cell culture models. They are commonly handled under atmospheric oxygen conditions (19–21% O_2_), although the physiological oxygen conditions are significantly lower in cerebrospinal fluid (CSF) (7–8% O_2_). We aimed to characterize oxygen levels in a Streptococcus (S.) suis-infected BCSFB model with transmigrating neutrophils. A BCSFB model with human choroid plexus epithelial cells growing on transwell-filters was used. The upper “blood”-compartment was infected and blood-derived neutrophils were added. *S. suis* and neutrophils transmigrated through the BCSFB into the “CSF”-compartment. Here, oxygen and pH values were determined with the non-invasive SensorDish^®^ reader. Slight orbital shaking improved the luminescence-based measurement technique for detecting free oxygen. In the non-infected BCSFB model, an oxygen value of 7% O_2_ was determined. However, with *S. suis* and transmigrating neutrophils, the oxygen value significantly decreased to 2% O_2_. The pH level decreased slightly in all groups. In conclusion, we characterized oxygen levels in the BCSFB model and demonstrated the oxygen consumption by cells and bacteria. Oxygen values in the non-infected BCSFB model are comparable to in vivo values determined in pigs in the CSF. Infection and transmigrating neutrophils decrease the oxygen value to lower values.

## 1. Introduction

Due to the global increase in antibiotic-resistant bacteria and thereby associated complications in treating certain bacterial infections [1,2], a need to identify new treatment opportunities exists. Therefore, research to understand the host–pathogen interaction is important. Besides in vivo studies, infection studies with cell culture models can reflect the (patho-) physiological situation [3]. A better understanding of patho-mechanisms may lead to identifying potential ways for alternative therapies. The effect and efficiency of such new therapies can be analyzed in physiologically relevant cell culture systems.

Therefore, suitable and valid physiologically relevant replacement methods are needed. Several cell culture systems have been established in the last years, including organoids, organ-on-a-chip, and 2D and 3D cell culture systems [4,5,6]. However, most in vitro cell culture models are commonly cultivated under atmospheric oxygen conditions in cell incubators (19–21% O_2_), although the physiological oxygen conditions in vivo are significantly lower in most tissues [7]. Especially during acute inflammation or infection, oxygen levels can locally decrease to hypoxic levels. These differences should be considered critically, as unphysiological oxygen levels in in vitro experiments can lead to altered phenotypes and gene expressions [8,9]. Furthermore, oxygen influences the reaction of immune cells [10,11,12]. Therefore, unphysiological oxygen levels can consequently lead in vitro to results that are not reflecting the in vivo situation. It was reviewed for neurophysiological studies that oxygen influences the metabolism and cell differentiation. Therefore, it is important to closely mimic in vivo oxygen levels [13].

An important disease in the neurophysiological environment is the inflammation of the meninges, induced by mainly invading bacteria and infiltrating neutrophils. Meningitis in humans and animals is still leading worldwide to high fatalities. *Streptococcus* (*S*.) *suis* is a zoonotic Gram-positive bacterium resulting in meningitis in humans and pigs [14,15]. In the absence of antibiotics, a mortality rate of up to 20% in pigs can be observed [16]. Humans with intensive and close contact with pigs or wild boars can be infected with *S. suis* and suffer from meningitis [17]. Whereas in pigs, worldwide, *S. suis* infections lead to high economic losses, outbreaks in humans are mainly reported from Asian countries [18,19]. The host–pathogen interaction during this disease is poorly understood. However, it is known that *S. suis*, like other bacteria, can enter into the cerebrospinal fluid (CSF) via the blood-CSF barrier (BCSFB), leading finally to inflammation of the meninges [14,17,20,21].

The BCSFB is formed by the choroid plexus epithelial cells and fenestrated endothelium. This blood–brain barrier is located inside the CSF-filled ventricles of the brain [22,23]. The BCSFB is described in several diseases of the brain as an important key structure [24]. Different cell culture systems of the BCSFB exist [25], and one model is formed by choroid plexus epithelial cells (HIBCPP) derived from a human tumor [26]. In this model, the cells are grown inverted on the outer side of filter inserts. Therefore, this model mimics the BCSFB with the physiological orientation of the cells, allowing manipulation from the basolateral side [27]. In a following step, this model was adapted by our group to analyze the host–pathogen interaction during the early phase of an *S. suis* meningitis. Blood-derived human neutrophil granulocytes were analyzed for the release of neutrophil extracellular trap (NETs) in the *S. suis*-infected “CSF” compartment [28]. NETs are one defense mechanism of the host to combat bacterial infections [29] and are discussed as one potential therapy opportunity. Interestingly, it was described that low oxygen concentrations (hypoxic conditions) influence NET formation as well as cell activity [10].

Our group recently determined the dissolved oxygen in CSF of pigs with and without meningitis in vivo [30] with the aim of using these in vivo data for validating in vitro tools. In the in vivo project, we were able to show that the physiological oxygen value in CSF is between 7–8% O_2_ (47–63 mmHg). The oxygen value was constant and not significantly influenced by *S. suis* infection and infiltrating immune cells during the early phase of meningitis in the CSF. However, no data exist yet concerning the oxygen value in the BCSFB cell culture model under infected and non-infected conditions.

The aim of this study was to implement a luminescence-based measurement technique to monitor the oxygen and the pH level in the “CSF” compartment of the *S. suis*-infected BCSFB cell culture model. Furthermore, the values were measured without infection. With this study, a further step can be made towards achieving an improved BCSFB model with and without infection mimicking the host–pathogen interaction during meningitis.

## 2. Materials and Methods

### 2.1. Streptococcus suis Cultivation

*Streptococcus suis* strain 10 (*S. suis* 10) is a virulent wild-type serotype 2 strain (*mrp*^+^, *epf*^+^, *sly*^+^) of multilocus sequence type (ST) 1 and was isolated from a pig with pneumonia [31,32]. Pigs infected intranasally or intravenously with this strain suffer from meningitis [33]. *S. suis* was cultivated on Columbia Agar with 7% Sheep Blood (Thermo Scientific ™ PB5008A). Serial dilutions were plated in duplicate, and colonies were counted after a 24-h incubation period at 37 °C to determine the colony-forming units per mL (CFU/mL). Working cryostocks of bacteria were generated by growing the bacteria to the exponential growth phase (OD_600nm_ = 0.6) in Todd Hewitt Broth (THB) (Becton, Dickinson and Company, Sparks Glencoe, MD, USA) at 37 °C without CO_2_. Aliquots with a final 15% Glycerol (Sigma Aldrich Corp., 13487-2, St. Louis, MO, USA) were frozen in liquid nitrogen and stored at −80 °C and used only once after thawing.

### 2.2. Purification of Human Neutrophils

In agreement with the local ethical board (permit no. 3295-2016, Hannover Medical School, Hannover, Germany), neutrophils were isolated from the blood (lithium-heparin) of healthy volunteers as previously described [34]. Briefly, neutrophils were isolated using the Polymorphprep system (Progen; 1.113 g/mL).

### 2.3. Two-D Model of the BCSFB with S. suis Infection and Neutrophil Transmigration

**Figure A1:**
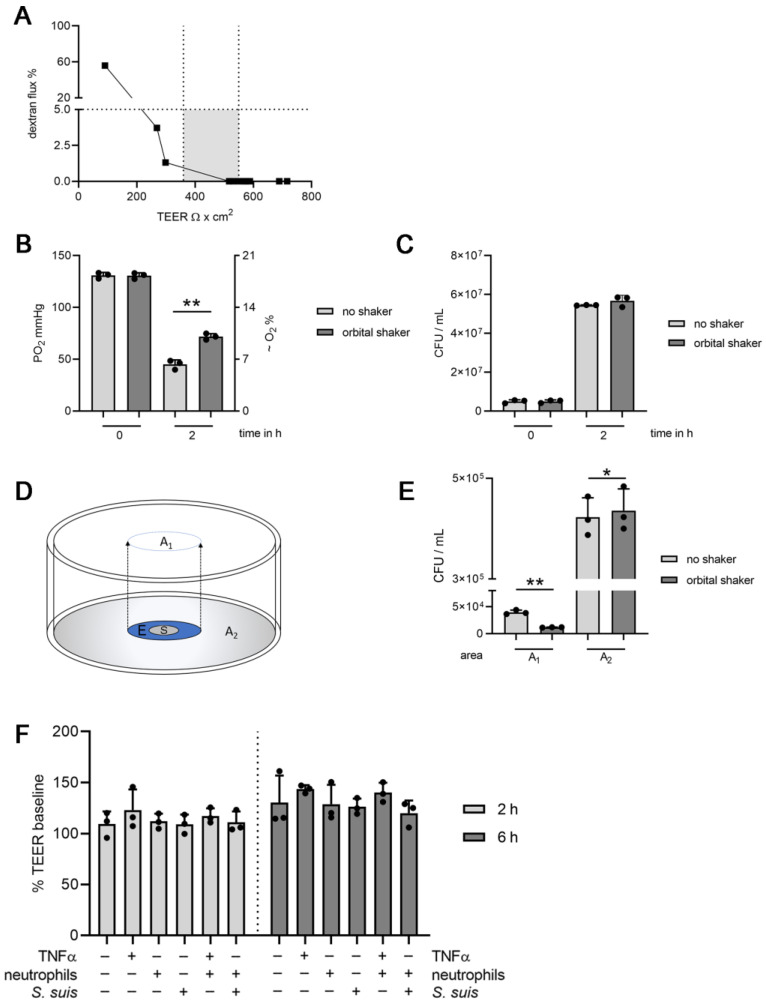
A shaking setup of the BCSFB model is obligatory to reduce bacterial-related false reduced oxygen levels inside the CSF compartment. (**A**) The dextran flux rate was determined as an indicator of barrier integrity. No cells on the filter (TEER = 0 Ω x cm^2^) led to a dextran flux rate of 100%. With increasing TEER values, the dextran flux rate decreased. Dextran flow rates lower than 5% indicate increasing barrier integrity of the HIBCPP layer. At the start of all experiments, only filters with TEER values of the experiment in the gray marked range were included. (**B**) The oxygen partial pressures were compared in a non-shaking and shaking setup during *S. suis* infection. After a 2-h incubation period with *S. suis*, significantly lower oxygen partial pressure was measured in the non-shaking setup (** *p* ≤ 0.01). (**C**) The determined bacterial number remained constant between the two setups. (**D**) Two areas were defined inside the well to quantify *S. suis* around the sensor spot (S) and in the outer circle. The area of “S” and a defined extra area = “E” were named “A1”. The rest of the well was named “A2”. (**E**) Significant different numbers of *S. suis* were reisolated from the two defined areas with the two shaking setups. The total amount of bacteria was comparable (* *p* < 0.05). (**F**) The TEER was not influenced by the shaking setup during an infection experiment with transmigrating neutrophils.

The BCSFB model with human choroid plexus epithelial cells (HIBCPP) was previously described and characterized [26,27], and further modified for infection studies with *S. suis* and transmigration of human neutrophils by our group [28]. Briefly, HIBCPP cells (passage 22–35) were grown on inverted translucent ThinCert™Cell Culture Inserts for 24 well plates (3 µm pore diameter, Greiner Bio One International GmbH, Kreismünster, Austria). An upper compartment (“blood” side in vivo) and a lower compartment (“CSF” side in vivo) were separated by the HIBCPP cell layer. Barrier integrity was determined by measuring transepithelial electrical resistance (TEER) using the Millicell^®^ ERS-2 (Merck Millipore Corp., Billerica, MA, USA) with the STX01 electrode. The TEER of each filter was calculated by multiplying the resistance value (Ω) with the area of the membrane (cm^2^) [35]. Based on the measurement of dextran flux through the barrier, in a similar way as previously described [28], a value of dextran flux lower than 5% matching a TEER in a range of 360–550 x Ω cm² was defined for filters with barrier integrity (Appendix A, Figure A1A) and used for all experiments.

The infection of the HIBCPP and transmigration of neutrophils were conducted as previously described with 1% heat-inactivated fetal calf serum (FCS) [28]. *S. suis* infection of the upper compartment was conducted for two hours with 8 × 10^6^ CFU/mL. For uninfected samples, cell culture medium only was used. After two hours of infection, the medium in all upper compartments was replaced by medium with or without human neutrophil granulocytes (1.6 × 10^6^/per well in 500 µL cell culture medium) for four hours (total experiment lasting six hours). As a transmigration control, human tumor necrosis factor alpha (TNFα) (20 ng/mL) was added to the lower compartment. The lower compartment (well) was filled with 1 mL medium. The cell culture was incubated at 37 °C and 5% CO_2_ with an air humidity of 100%.

### 2.4. Profile of Bacterial Growth in the 2D-Infection Model

The local bacterial distribution in the wells—possibly leading to different dynamic quenching quantities within the sensor matrix for oxygen and pH determination—was investigated by comparing a shaking and non-shaking setup. For shaking, the plate was placed during incubation on an orbital shaker with a slow rotation of 15 rpm (RS-OS 5; Phoenix Instrument GmbH, Garbsen, Germany). The filters were inserted in 24 well plates (see above) without HIBCPP cells, and *S. suis* was pipetted into the upper compartment. After incubation at 37 °C and 5% CO_2_ with an air humidity of 100%, both well plates (shaken and non-shaken) were carefully refrigerated at 4 °C to stop any further bacterial growth in the wells. Within the following 18 h in the refrigerator, the bacteria settled to the bottom of the wells, partially projecting the distribution gradient onto the surface. The medium was carefully sucked off without changing the bacterial distribution. The bacterial amounts in two defined areas on the bottom of the wells were completely picked up with a sterile eyelet. The surface around the oxygen sensor spot position on SDR plates (A_1_) was extended by a few millimeters to a defined radius of r = 0.245 cm and a corresponding area of 0.19 cm^2^. The remaining surface area of the well (A_2_) therefore amounted to 1.71 cm^2^. To compensate for the different area sizes, the eyelet samples were dissolved in different PBS volumes with a ratio of 1:9 (V_1_ = 0.5 mL; V_2_ = 4.5 mL) and were thoroughly homogenized to determine the CFU/mL by plating serial dilutions on blood agar plates.

### 2.5. Read Out: S. suis and Neutrophils in Lower (“CSF”) Compartment

The CFU/mL was determined in the upper compartment at timepoint 0 h. After two and six hours the CFU/mL was determined in the lower compartment as described above.

The number of transmigrated neutrophils was determined by flow cytometry. Therefore, 1 mL from the lower compartment was fixed with paraformaldehyde (final 4%; Science Services GmbH, Munich, Germany). The fixed cells were analyzed based on forward (FSC) and sideward (SSC) scatter using Attune^®^ NxT Acoustic Focusing Flow Cytometer (Invitrogen Corp., Carlsbad, CA, USA).

### 2.6. Read Out: Oxygen and pH Value in the Lower (“CSF”) Compartment

Oxygen and pH values were measured at the beginning of the experiment (0 h post infection), after two hours of *S. suis* infection (2 h post infection), and after four additional hours with interacting neutrophils (6 h post infection). An SDR SensorDish^®^ Reader Basis-Set (PreSens GmbH, Regensburg, Germany) in combination with one additional extension set was used. For the parallel recording of oxygen and pH levels during the transmigration experiments, the selected HIBCPP filter was transferred to an OxoDish^®^ OD24 or HydroDish^®^ HD24 well plate (PreSens GmbH, Regensburg, Germany) with integrated oxygen or pH sensors.

All experiments were performed with an equilibrated medium of about 18% O_2_. Due to the upregulated CO_2_ concentration and the high humidity in classical cell incubators, the amount of O_2_ was less than in dry air (20.95%) [36]. Therefore, the respective wells of the SDR plates were prefilled with a medium that was incubated for several hours in the atmosphere of the incubator at 37 °C and 5% CO_2_ with 100% humidity. Wells without oxygen-consuming factors but with equilibrated medium served as positive controls (100% O_2_ sat) during the time of the experiment, whereas specially prepared negative samples were characterized by a continuous 0% O_2_ saturation.

The measurements were performed inside the incubator at 37 °C and 5% CO_2_. To maintain a continuous temperature of 37 °C during working steps outside the incubator (lower temperature), all work was carried out on a 37 °C heating plate (CultureTemp^®^; Bel-Art Products-SP Scienceware, Inc., Wayne, NJ, USA). 

Since the oxygen concentration in liquid media depends on the ambient temperature, 37 °C was used as a basis for calculating the current oxygen content in the samples at different timepoints.

The dependence of the oxygen concentration in the liquid medium on the surrounding pressure of the atmosphere was considered by measuring the ambient air pressure in the incubator for each experiment (barometer DK323 HumiBaroLog Plus; Driesen+Kern GmbH, Bad Bramstedt, Germany) as recommended in recent literature [36]. The actual barometric conditions were compensated by the SDR software for the oxygen calculation.

The oxygenation of the samples was read in the unit mmHg (torr). In addition to the partial pressure, the amount of oxygen is also presented in the relative unit volume percent (%) that depends on the current barometric pressure. Since the air pressure itself depends on the altitude and the current weather conditions, relative units such as O_2_ volume percent are therefore less accurate than the partial pressure reading.

For the oxygen measurements, all samples were in duplicate, and the results were averaged, whereas the pH values were only determined in unique items.

OxoDish^®^ OD24 (PreSens GmbH) well plates were placed in the cell incubator either without any movement or on an orbital shaker with the slow rotation of 15 rpm (RS-OS 5; Phoenix Instrument GmbH). 

### 2.7. Filter Immunostaining of LL-37 and Myeloperoxidase

After washing the filters, the membrane was cut out and stained for LL-37. The filters were stained floating in 250 µL liquid and washed in 500 µL liquid. The filters were blocked and permeabilized for 60 min in PBS containing 10% goat serum and 0.5% TritonX-100. Incubation of antibodies was conducted in PBS containing 2% BSA. The filters were incubated with a polyclonal mouse-anti-LL-37 antibody (1:50, Hycult biotech b.v., Uden, the Netherlands) combined with a polyclonal rabbit anti-myeloperoxidae antibody (1:300, Dako GmbH, Jena, Germany) for 1 h at room temperature. After washing steps, AlexaFluor 488 goat anti-mouse (1:500, Molecular Probes) and AlexaFluor 633 goat anti-rabbit (1:500, Invitrogen) were used as secondary antibodies. Together with the secondary antibodies, staining of the cytoskeleton with phalloidin Alexa 546 (1:100, Invitrogen, Carlsbad, CA, USA) and the DNA with Hoechst (1:250,000, Invitrogen) was conducted for 45 min at room temperature. After staining, the filters were washed and embedded in ProLong Gold antifade without DAPI (Invitrogen). Samples were recorded using a Leica TCS SP5 confocal inverted-base fluorescence microscope with an HCX PL APO 40× 0.75–1.25 oil immersion objective lens. Settings were adjusted with control preparations using isotype control antibodies.

### 2.8. Statistical Analyses

All experiments were performed on three separate occasions. Data were analyzed using Excel 2019 (Microsoft) and GraphPad Prism 9.1.0 (GraphPad Software). Differences were analyzed as stated in the figure legends. The significance is indicated as follows: * *p* ≤ 0.05, ** *p* ≤ 0.01, *** *p* ≤ 0.001, and **** *p* < 0.0001.

## 3. Results

### 3.1. Establishing Oxygen Measurement Inside the CSF Compartment of the BCSFB Model during S. suis Infection under Shaking Conditions

**Figure 1:**
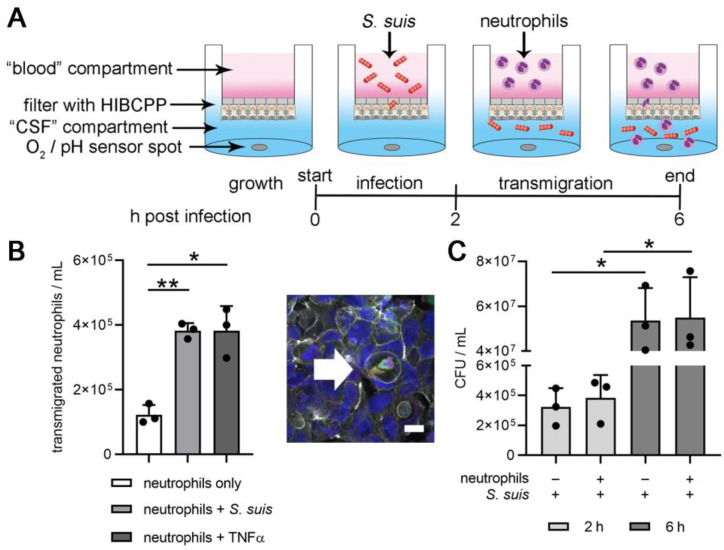
Experimental setup of the BCSFB model under shaking conditions. (**A**) The experimental setup of the BCSFB model is displayed. Human choroid plexus epithelial cells (HIBCPP) form the BCSFB barrier and divided the model into an upper “blood” compartment and a lower “CSF” compartment. Sensor spots for oxygen and pH measurement are inside the CSF compartment. (**B**) The number of transmigrated neutrophils was quantified inside the CSF compartment at the end of the six-hour experiment. The shaking setup did not influence the transmigration. One example of an immunofluorescence microscopy picture of the choroid plexus cells grown on a filter shows a transmigrating neutrophil (arrow). Scale bar 10 µm; blue = DNA, gray = phalloidin, green = LL-37, red = myeloperoxidase; single channels and overview pictures are presented in Figure A3. (**C**) The host–pathogen interaction of neutrophils and *S. suis* were not influenced regarding the neutrophil killing capacity of *S. suis*. All data are presented as mean ± SD of three independent experiments. Statistical differences were analyzed with one-tailed paired Student’s *t*-test (* *p* < 0.05, ** *p* < 0.01).

To enable oxygen measurements in the BCSFB model, the classical culture model that had been previously described [27,28] was modified. First, the 24-well-plate was exchanged for a plate containing oxygen and pH sensor spots for real-time measurement during the experiment (Figure 1A). As a control experiment, the optimal gas exchange in the medium with the presence of bacteria was determined under non-shaking compared to shaking conditions, since the previously described system had only been established under non-shaking conditions. Here, we strived for a shaking condition to allow optimal gas exchange and improved bacterial distribution. As the sensor spots are located at the bottom of the well and transmigrated bacteria gravitate to the bottom, oxygen was compared under non-shaking and orbital shaking conditions and significantly lower oxygen values were revealed under non-shaking conditions two hours post infection (Figure A1B). These significantly lower oxygen values cannot be explained by differences in the total CFU/mL inside the CSF-compartment (Figure A1C). Nevertheless, the distribution of bacteria when comparing the non-shaking and shaking system was different. Significantly fewer bacteria were recovered from the area of the sensor spot under shaking conditions (Figure A1D–E). Therefore, this microenvironment with higher bacterial numbers can explain the identified lower oxygen level in the non-shaking setup. The shaking setup was therefore used in all following experiments. 

To verify whether the shaking setup enabled the studying of the host–pathogen interaction of neutrophils and *S. suis* after transmigration inside the CSF compartment, neutrophils/mL and CFU/mL were determined. A significantly higher number of neutrophils transmigrated after stimulation with *S. suis* or TNFα compared to non-stimulated neutrophils (Figure 1B), a phenotype that is similar to the described model in the absence of the shaking setup [28]. Furthermore, the CFU/mL increased over time and was not significantly different in the presence or absence of neutrophils. This again confirms results from the previously published data with non-shaking conditions (Figure 1C) [28]. Indeed, neutrophils counteract against *S. suis* with different defense mechanisms. However, in the absence of plasma and efficient opsonization, neutrophils mainly act bacteriostatic by inhibiting the growth of the bacteria, rather than bactericidal by killing the bacteria. In addition, the barrier integrity determined by TEER increased during the experiment as previously observed and did not significantly differ between the different stimulations (Figure A1F) [28]. In summary, the shaking setup did not significantly affect barrier integrity, neutrophil transmigration, or growth of *S. suis* inside the CSF compartment and was useful in the described context. 

### 3.2. Choroid plexus Epithelial Cells (HIBCPP), Neutrophils and S. suis Consume Oxygen in the BCSFB Model

**Figure 2:**
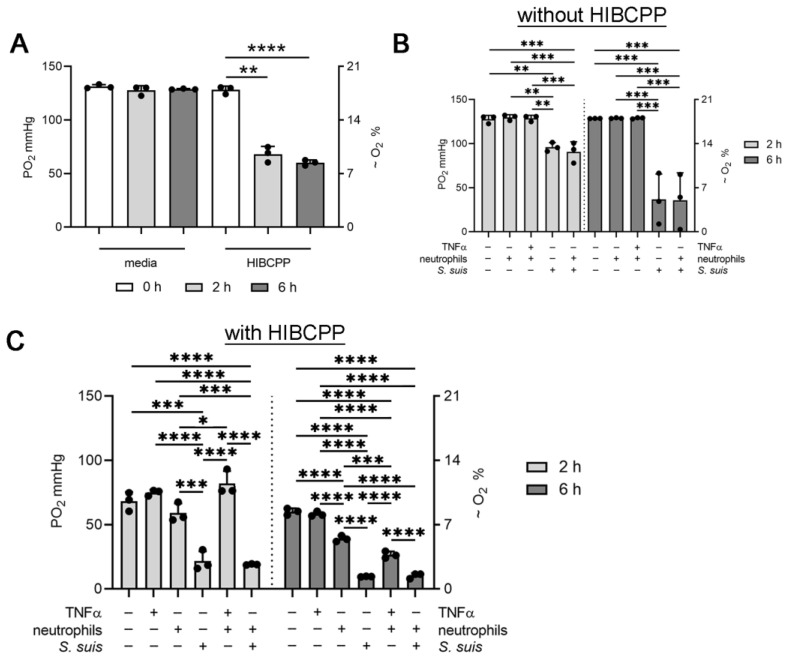
Choroid plexus epithelial cells (HIBCPP), neutrophils, and *S. suis* consume oxygen over time. The oxygen level was determined in the CSF compartment in a shaking setup. (**A**) The oxygen level did not change in media, whereas with HIBCPP cells, the oxygen decreased over time. (**B**) Non-activated neutrophils did not consume a high level of oxygen, whereas *S. suis* significantly consumed oxygen. (**C**) The host–pathogen interaction of HIBCPP cells, transmigrating neutrophils, and *S. suis* significantly consumed oxygen. All data are presented as mean ± SD of three independent experiments. Statistical differences were analyzed in A with a one-tailed paired Student’s *t*-test in each group at 0 h. Statistical differences were analyzed in B and C with a one-way ANOVA at each time-point (*p* < 0.0001), followed by Tukey multiple comparisons (* *p* < 0.05, ** *p* < 0.01, *** *p* < 0.001, **** *p* < 0.0001).

The shaking setup was now used to determine the oxygen level and oxygen consumption of all interaction partners (HIBCPP, neutrophils, and *S. suis*) inside the CSF compartment. As all interaction partners are possible oxygen consumers, the oxygen level was determined first in single setups, and finally with all interaction partners in one setup. The oxygen level was significantly decreased by HIBCPP cells from 128 ± 3.5 mmHg at the beginning of the experiment, to 68 ± 7.2 mmHg after two hours, and 60 ± 2.6 mmHg after six hours (Figure 2A). Therefore, the HIBCPP cells were identified as oxygen consumers (Figure 2A). Inside a system without HIBCPP cells, neither unstimulated neutrophils nor TNFα stimulated neutrophils consumed oxygen. A significant decrease in the oxygen level was detected in *S. suis*-infected wells as well as in the presence of *S. suis*-infected neutrophils. After two hours, the oxygen level was decreased to 96 ± 5.1 mmHg (*S. suis*) and 91 ± 11.5 mmHg (*S. suis*-infected neutrophils). After six hours, the oxygen level decreased further to 37 ± 29 mmHg (*S. suis*) and 36 ± 31 mmHg (*S. suis*-infected neutrophils). Therefore, under this chosen experimental setup, *S. suis* is an essential oxygen consumer over time, whereas neutrophils consume less oxygen (Figure 2B).

Inside a system with HIBCPP cells, transmigrating neutrophils significantly consumed oxygen after six hours (27 ± 2.6 mmHg). A two-hour value was determined as the baseline, since based on the experimental setup, neutrophil transmigration started first after two hours. These data are in good accordance with the data published in [37]. The authors show that activated transmigrating PMN rapidly depleted microenvironmental oxygen in the presence of intestinal epithelial. In this study, it was demonstrated that activated PMN are able of depleting local oxygen to a level that neighboring epithelial cells “sense” hypoxia and stabilize the HIF transcriptional machinery. Such localized oxygen depletion was critical for effective mucosal protection and inflammatory resolution during acute colonic inflammation.

Importantly, a significant decrease in the oxygen level was detected in the CSF compartment of *S. suis*-infected HIBCPP cells. After two hours, the oxygen level was decreased to 21 ± 7.7 mmHg (*S. suis*). After six hours, the oxygen level decreased further to 10 ± 0.3 mmHg (*S. suis*). An equal value was detected in the presence of transmigrated neutrophils and *S. suis* 10 ± 2.1 mmHg. Therefore, it may be concluded that transmigrated neutrophils consume more oxygen than non-transmigrating neutrophils. Infection with *S. suis* and transmigrating neutrophils significantly decrease the oxygen values in the CSF compartment of the BCSFB model (Figure 2C).

### 3.3. S. suis and Neutrophils Slightly Decrease the pH in the BCSFB Model

**Figure 3:**
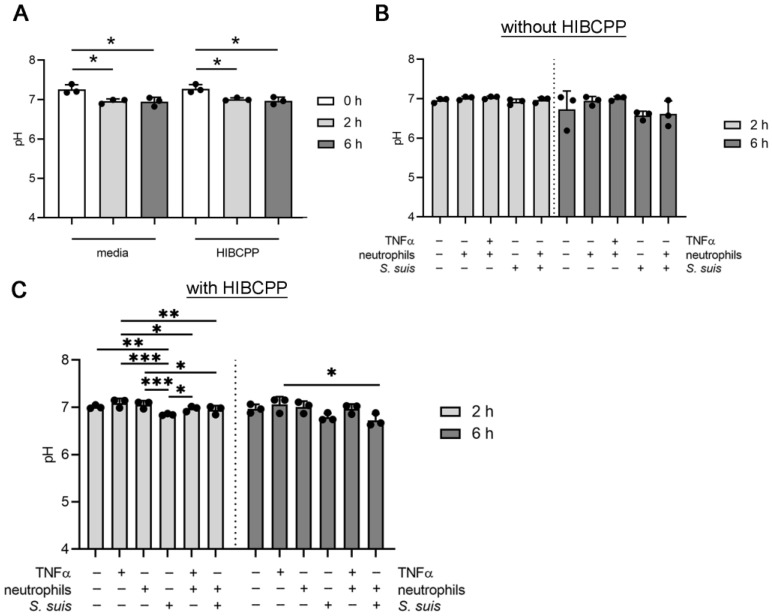
The pH in the CSF compartment is slightly decreased by *S. suis* and interacting neutrophils after a six-hour incubation period. The pH level was determined in the CSF compartment in a shaking setup. (**A**) The pH decrease in media and with HIBCPP cells is comparable. (**B**) No significant influence on pH was detected in the absence of HIBCPP cells. (**C**) The host–pathogen interaction of HIBCPP cells, transmigrating neutrophils, and *S. suis* significantly decreased the pH slightly after six hours. All data are presented as mean ± SD of three independent experiments. Statistical differences were analyzed in A with a one-tailed paired Student’s *t*-test in each group at 0 h. Statistical differences were analyzed in B and C with a one-way ANOVA at each time-point (**C**: *p*_2h_ = 0.002; *p*_6h_ = 0.02), followed by Tukey multiple comparisons in (**C**) (* *p* <0.05, ** *p* < 0.01, *** *p* < 0.001.

Alongside determining the oxygen level in the BCSFB model, the influence on the pH of all interaction partners (HIBCPP, neutrophils, and *S. suis*) inside the CSF compartment was determined. Like the oxygen level, the pH value was measured first in single setups, and finally with all interaction partners in one setup. The pH level was significantly decreased by HIBCPP cells from pH = 7.3 ± 0.10 at the beginning of the experiment, to pH = 7.0 ± 0.04 after two hours, and pH = 7.0 ± 0.10 after six hours (Figure 3A). However, the same effect was detected only with media. Therefore, the HIBCPP cells do not influence the pH (Figure 3A). Inside a system without HIBCPP cells, neither neutrophils nor *S. suis* significantly decreased the pH (Figure 3B). Inside a system with HIBCPP cells, transmigrating neutrophils did not influence the pH after six hours (pH = 7.0 ± 0.16). A significant decrease in pH was detected in the CSF compartment of *S. suis*-infected HIBCPP cells. After two and six hours, the pH level was decreased to 6.8 ± 0.08 (*S. suis* 6 h). A comparable but lower value was detected in the presence of transmigrated neutrophils and *S. suis* (6.7 ± 0.1 mmHg). Therefore, *S. suis* and neutrophils infected with *S. suis* slightly decrease the pH value in the CSF compartment of the BCSFB model (Figure 3C).

## 4. Discussion

In the last years, an increasing numbers of publications have demonstrated how important oxygen levels are for cells in cell cultures [38], including cell cultures from the central nervous system [39,40,41]. To allow reproducibility of results reported from cell culture experiments, there is a need to report oxygen values [42]. Furthermore, the cultivation of cell culture systems under physiologically relevant oxygen values would lead to an optimal improvement to mimic the in vivo situation. Mostly, cell cultures are incubated in incubators with higher oxygen values than those values that are physiologically relevant. This oxygenation is termed differently, called both supraphysiological oxygen level and hyperoxic oxygen level [38,42]. Several studies have demonstrated that hyperoxic oxygen levels influence cells. Depending on the oxygen values, the mitochondrial function in neurons differs in its reaction to HIV viroproteins [43]. Neutrophils incubated under oxygen concentrations of 1% O_2_ (7 mmHg) compared to 21% O_2_ (159 mmHg) significantly release fewer NETs [10]. Furthermore, hypoxia is described as modulating the immunometabolism, and therefore, oxygen can influence the host–pathogen interaction [44].

Recently, we established and evaluated an in vivo measurement system to characterize the oxygen level in parallel with bacterial numbers (CFU/mL), the cell number, and pH level inside the CSF of healthy compared to *S. suis*-infected pigs [30]. Infected animals were anesthetized over a seven-hour period with isoflurane in air/oxygen at physiologic arterial partial pressure of oxygen. Interestingly, the detected partial pressure of oxygen in the CSF in vivo remained constant in a range of 47–63 mmHg, reflecting 7–8% oxygen. Importantly, this value was independent of the infection status (bacterial or cell number) during the early phase of meningitis, since regulatory mechanisms of the host inside the brain might help to control and stabilize pO_2_ at least during the onset of meningitis. However, knowledge of this physiologically relevant in vivo oxygen level in the CSF might help to improve in vitro systems.

In good correlation to the in vivo data, here in this study, we present the establishment of a real-time oxygen and pH measurement system, and the status quo oxygen and pH characteristics inside an *S. suis*-infected BCSFB model with transmigrating neutrophils that closely mimics the in vivo situation during meningitis. This model was already used for infection studies with typical meningitis-inducing bacteria like *S. suis* [28] or *Neisseria meningitidis* [27,45]. In summary, we were able to show that the consumption of oxygen by HIBCPP cells under non-infection status (Figure 2A) reflects the recently published physiologically relevant oxygen values in vivo [30].

Neutrophils and *S. suis* decreased the oxygen level to less physiologically relevant oxygen levels at 6 h after infection in vitro (Figure 2). This result contrasts with the data seen in vivo where this value was independent of the infection status (bacterial or cell number) during the early phase of meningitis [30]. For ethical reasons to reduce the psychological burdens of animals, a later phase of meningitis was not studied in vivo. Thus, it might be speculated that during later severe meningitis, lower oxygen values might also be detectable in vivo as seen in the intestine when transmigrated neutrophils shape the microenvironment [37].

**Figure A2:**
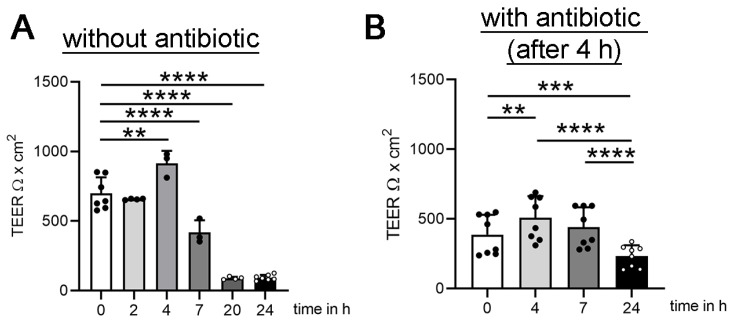
*S. suis* significantly decreased the barrier integrity after seven hours. The TEER values of *S. suis*-infected HIBCPP filters were measured before infection (0 h) and after 4, 7, 20, and 24 h (h). The filters were infected and incubated for 24 h with and without antibiotic treatment. (**A**) A significant decrease in TEER was detectable after 7 h *S. suis* infection in the absence of antibiotics. After 20 h, the low TEER reflected a loss of barrier integrity. (**B**) Antibiotic treatment after 4 h did not lead to a TEER value after 24 h in a range higher than 380 Ω x cm^2^. The mean value ± SD of independent experiments is presented. Each dot represents one filter. Statistical differences were analyzed with one-way ANOVA (*p* < 0.0001), followed by Dunnett’s multiple comparison test in A and Tukey’s multiple comparison test in B, (** *p* < 0.01, *** *p* < 0.001, **** *p* < 0.0001).

**Figure A3:**
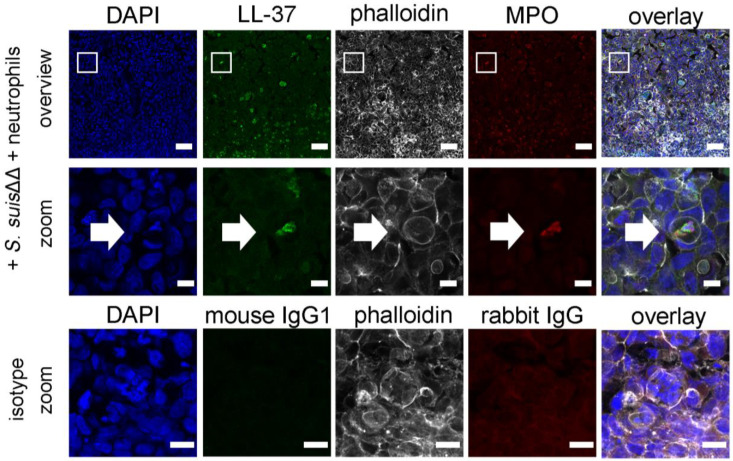
LL-37 and myeloperoxidase signals identify a transmigrating neutrophil through the BCSFB. Immunofluorescence microscopy was conducted to detect transmigrating neutrophils. Filters were stained and imaged from the “CSF” side. White squares highlight the area of magnification. Settings were adjusted to the respective isotype control; blue = DNA, green = LL-37, red = myeloperoxidase (MPO) and gray = phalloidin). Scale bar overview = 50 µm; scale bar zoom = 10 µm.

However, in our described in vitro model, a drop in oxygen was already detectable at 6 h upon infection. Thus, implementing an oxygen supply system in the existing model might be considered. It is challenging to deliver oxygen to cell cultures at a physiologically relevant level [36]. Creative approaches are needed if 2D and 3D cell cultures need to reflect two different compartments with different oxygen levels in vivo as for example, CSF and blood compartment in this study. Possibilities are a system with a floating medium or one with oxygen-permeable culture plates. Research approaches involving infection studies with, for example, bacteria as oxygen consumers are suboptimal and will need improvements in the future. Our described in vitro model runs under six hours’ infection, as after seven hours, the TEER, as a measurement parameter for barrier integrity, decreased (Figure A2A). To reduce the cytotoxic overgrowth of *S. suis* inside the BCSFB model, an antibiotic treatment was also tested in control experiments. The antibiotic treatment was tested after four hours to decrease the number of *S. suis* and reduce the production of the pore-forming toxin Suilysin [46,47]. However, the TEER again dropped over 24 h (Figure A2B). As antibiotics can influence host–cell interaction as, for example, neutrophil functions, we aimed to establish a host–pathogen interaction of neutrophils and *S. suis* without antibiotic treatment. Thus, the experimental setup was fixed for six hours’ interaction of host cells and *S. suis* (Figure 1A). Immunofluorescence microscopy confirmed transmigrating neutrophils through the BCSFB by neutrophil-specific stainings of, for example, LL-37 and myeloperoxidase in this model during oxygen measurements (Figure A3).

In summary, the limitations of a longer investigation period with this setup is indicated by several points: 1. the life-span of isolated neutrophils [48,49], 2. the growth of *S. suis*, leading to values higher than in vivo determined CFU/mL (~10² higher CFU/mL inside the CSF of the BCSFB model compared to *S. suis* CFU/mL inside the CSF of piglets in vivo [30]), 3. the decrease in barrier integrity after six hours, and 4. the decrease in oxygen leading to values lower than determined in vivo [30].

However, the BCSFB setup is a valid system to characterize host–pathogen interaction during the early phase of meningitis and to investigate, for example, new treatment strategies that must pass the BCSFB in vivo, especially without conditions mimicking infectious processes. Further studies might help to develop a more complex system with the permanent exchange of media, therefore enabling an exchange of bacteria and neutrophils after transmigration in the CSF compartment [25]. Such systems are costly for studying host–pathogen interactions, especially when contaminated with bacteria, since each time the tubing systems need to be replaced to avoid cross-contamination of individual samples. Therefore, the trans-well system presented here is definitely an easy-to-use alternative to mimic the early phase of meningitis. One limitation is that the presented model still relies on the use of FCS, which somewhat defeats the purpose, although the FCS was already decreased during the experimental setup to only 1% FCS (see Material and Methods and de Buhr et al., 2017). In the future, work needs to be done to allow the usage of human serum and potential growth factor supplementation as a replacement for fetal calf serum. Overall, the availability, characterization, and optimization of cell culture systems as described here that present the physiological situation can help to keep animal experiments to a minimum. 

## 5. Conclusions

In conclusion, we characterized oxygen levels in a non-infected and infected human BCSFB model with transmigrating human neutrophils. This reflects the in vivo situation during meningitis. We demonstrated the oxygen consumption by host cells and bacteria in this model. Whereas oxygen values in the non-infected BCSFB model are comparable to in vivo values determined in pigs in the CSF, infection and transmigrating neutrophils decrease the oxygen value in the “CSF” compartment to lower values. Therefore, future studies should focus on establishing an in vitro oxygen supply to reach in vivo relevant oxygen conditions and further improve this cell culture model for research of the host–pathogen interaction during meningitis.

## Data Availability

The authors confirm that the data supporting the findings of this study are available within the published article. Raw data were generated at the Department of Biochemistry and Research Center for Emerging Infections and Zoonoses (RIZ), University of Veterinary Medicine Hannover, Hannover, Germany. Derived data supporting the findings of this study are available from the corresponding authors N.d.B. and M.v.K.-B. on request.

## Appendix A

In all graphs, the mean ± SD from three independent experiments is presented. Statistical differences were analyzed in B, C, and D with a one-tailed paired Student’s *t*-test in each time-point. Statistical differences were analyzed in E with a one-way ANOVA at each time-point, (* *p* <0.05).
